# Sputum microscopy for the diagnosis of HIV-associated pulmonary tuberculosis in Tanzania

**DOI:** 10.1186/1471-2458-8-68

**Published:** 2008-02-21

**Authors:** Mecky Matee, Lillian Mtei, Tarja Lounasvaara, Wendy Wieland-Alter, Richard Waddell, Johnson Lyimo, Muhammad Bakari, Kisali Pallangyo, C Fordham von Reyn

**Affiliations:** 1 Muhimbili University of Health and Allied Sciences, Dar es Salaam, Tanzania; 2 Tuberculosis Reference Laboratory, National Public Health Institute, Turku, Finland; 3 Infectious Disease and International Health, Dartmouth Medical School, Lebanon, NH, USA

## Abstract

**Background:**

In many resource poor settings only sputum microscopy is employed for the diagnosis of HIV-associated pulmonary tuberculosis; sputum culture may not be available.

**Methods:**

We determined the diagnostic accuracy of sputum microscopy for active case finding of HIV-associated pulmonary tuberculosis using TB culture as the reference standard.

**Results:**

2216 potential subjects screened for a TB vaccine trial submitted 9454 expectorated sputum specimens: 212 (2.2%) were sputum culture positive for *Mycobacterium tuberculosis *(MTB), 31 (0.3%) for non-tuberculous mycobacteria, and 79 (0.8%) were contaminated. The overall sensitivity of sputum microscopy was 61.8% (131/212) and specificity 99.7% (9108/9132). Sputum microscopy sensitivity varied from 22.6% in specimens with < 20 colony forming units (CFU)/specimen to 94.2% in patients with > 100 CFU/specimen plus confluent growth. The incremental diagnostic value for sputum microscopy was 92.1%, 1.8% and 7.1% for the first, second and third specimens, respectively. The positive predictive value and negative predictive values for sputum microscopy were 84.5% and 99.1%, respectively. The likelihood ratio (LR) of a positive sputum microscopy was 235.1 (95% CI 155.8 – 354.8), while the LR of a negative test was 0.38 (95CI 0.32 – 0.45). The 212 positive sputum cultures for MTB represented 103 patients; sputum microscopy was positive for 57 (55.3%) of 103 patients.

**Conclusion:**

Sputum microscopy on 3 expectorated sputum specimens will only detect 55% of culture positive HIV-infected patients in active screening for pulmonary tuberculosis. Sensitivity is higher in patients with greater numbers of CFUs in the sputum. Culture is required for active case finding of HIV- associated pulmonary tuberculosis.

## Background

Despite recent advances in mycobacteriology [1], early laboratory diagnosis of tuberculosis in the vast majority of disease-endemic countries remains microscopic examination of the stained sputum smear [2,3]. The standard WHO recommendation for TB diagnosis in the DOTS program is the use of direct sputum microscopy on 3 stained sputum specimens [4]. First and third are on spot, while the second is the early morning sample. Currently no other diagnostic tool, including sputum culture, is available which could be implemented affordably in resource poor settings, where the burden of disease is greatest.

However, even before the current HIV epidemic it was recognized that the sensitivity of sputum microscopy for the microbiologic confirmation of TB was limited. With HIV infection the sensitivity of sputum microscopy may be reduced even further because of the lower rate of caseation necrosis, and consequent lower numbers of AFB in the airway [5]. HIV may also reduce the specificity of sputum microscopy by increasing the proportion of patients with non-tuberculous mycobacteria [6]. Further, there is still a debate about whether two or three sputum specimens should be examined for diagnosis of TB [7-9]. Reducing the recommended number of specimens examined from three to two could benefit TB control programs by using fewer resources and by reducing the time spent on case detection [10]. In the present study we assessed the test characteristics of smear microscopy on three concentrated sputum specimens during active TB case finding among HIV-infected patients in Dar es Salaam, Tanzania.

## Methods

### Patients and study protocol

Participants were 2216 HIV-positive subjects screened for enrollment in the DARDAR Study between October 2001 and September 2006. DARDAR is a study on the epidemiology and vaccine-based prevention of HIV-associated tuberculosis [11] being conducted in Dar es Salaam, Tanzania. Eligibility required a CD4 count > 200, a BCG scar, and no evidence of active TB. At baseline, all potential subjects had a physical examination and standardized interview that included questions about weight loss in the past 3 months and about the presence and duration of any cough or fever. All subjects had a baseline chest x-ray and submitted 3 expectorated sputum samples for microbiologic testing. Subjects were instructed in the need for deep cough and asked to provide one spot sputum sample and to label and bring two first morning samples; subjects who were unable to produce a spot specimen were asked to bring three first morning samples. Subjects with active TB on baseline screening were not eligible for further study. Eligible subjects were started on the immunization protocol and were seen every 3 months and whenever new symptoms develop. At each visit subjects were re-evaluated for tuberculosis with physical examination, chest x-ray and collection of 3 expectorated sputum samples.

### Laboratory methods

Sputum samples were decontaminated using the modified Petroff's method and concentrated by centrifugation at 3000 g for 15 minutes. Smears were screened by auramine staining and positive smears were counterstained by the Ziehl Neelsen (ZN) staining technique without removing the auramine. Smears were read without knowledge of culture outcomes and results were categorized as 3+ (> 10 AFB/oil field), 2+ (1 – 10 AFB/oil field), 1+ (10 – 99 AFB/100 oil fields), scanty (1 – 9 AFB/100 oil fields) and negative (0 AFB/100 oil fields). For each smear, a total of 100 microscopic fields were examined as per protocol. Each sample was then cultured in both pyruvate and glycerol containing Lowenstein Jensen media at 37°C for up to 8 weeks. Plates were examined weekly for growth. Colonies were identified according to criteria based on the speed of growth and macroscopic features e.g. roughness and pigment production. Culture results were expressed as actual number of colonies (if less than 20 colonies/slant) 1+ (20–100 colonies/slant, 2+ (discrete innumerable colonies/slant) and 3+ (for confluent growth). Positive cultures were shipped to the US for confirmation by DNA probe (AccuProbe, San Diego, CA, USA).

### Quality assurance

Quality assurance was accomplished by assessing the quality and adequacy of specimens, and by monitoring microscopy and culture procedures, preparation and storage of reagents and performance of equipment against established laboratory operating procedures. Patients were requested to provide an additional specimen in case of submitting either an inadequate or salivary sample.

For smear microscopy, positive and negative control slides were included with each batch of new reagents and, in a blind manner, when reading patient smears. All slides were read independently by three experienced microscopists, and kept for up to three months for external quality control. Review of smear and culture results provided an internal quality assurance measure. Additional quality measures for cultures included monitoring of; quality of water, decontamination, digestion, and concentration procedures, inspissation and incubation temperatures, and measurement and adjustment of pH of culture media. A standard laboratory strain *M. tuberculosis *H37Rv was used as a positive control. Identification of mycobacteria was based on growth and colonial morphology and was confirmed by further testing of the isolates using DNA probes (Accuprobe; Gen-Probe).

Between 2001 and 2006 the study laboratory participated in the UK National External Quality Assurance Scheme for TB sputum microscopy and sputum culture, and had cumulative scores of 100% and 98% for SM and sputum culture, respectively.

### Ethics

The research protocol was approved by the Ethics Committee of the Muhimbili University of Health and Allied Sciences and the Dartmouth Committee for the Protection of Human Subjects.

### Statistical analysis

The sensitivity, specificity, positive and negative predictive values of the sputum smear examinations were calculated by using the sputum culture results as the "gold standard."

## Results

Between October 2001 and September 2006 a total of 2216 potential study subjects submitted 9454 expectorated sputum specimens for examination. Of the 9454 specimens, 212 (2.2%) were sputum culture positive for MTB, 31 (0.3%) were sputum culture positive for NTM, and 79 (0.8%) were contaminated (Table 1). DNA probes were available on 166 of 212 specimens and were uniformly positive (the remaining samples were either contaminated or not viable when received in the US for testing). Sensitivity and specificity of direct sputum microscopy were 61.8% and 99.7%, respectively based on total samples submitted. Sputum microscopy positivity varied from 22.6% in patients whose culture yielded < 20 CFU per slant to 94.2% in patients with > 100 CFU and confluent growth (Table 2). The positive predictive value (PPV) and negative predictive value (NPV) for direct SM were 84.5% and 99.1%, respectively based on total samples submitted. The likelihood ratio (LR) of a positive sputum microscopy was 235.1 (95% CI 155.8 – 354.8), while the LR of a negative test was 0.38 (95CI 0.32 – 0.45).

**Table 1:** Sputum microscopy and sputum culture results on all specimens

	SC positive	SC negative	**Total**
SM positive	131^1^	24	**155**
SM negative	81	9108	**9189**

**Total**	**212**^2^	**9132**	**9344**^3,4^

^1 ^103 patients

^2 ^DNA probes performed on 166 isolates (94 SM positive, 72 SM negative); 46 additional isolates considered MTB based on growth and culture morphology

^3 ^Excludes 79 culture contaminated and 31 NTM

^4 ^Test characteristics of SM:

**Sensitivity **= 131/212 = 61.8%(95% CI 54.9% -68.4%)

**Specificity **= 9108/9132 = 99.7% (95% CI 99.6% – 99.8%)

**Positive predictive value **= 131/155 = 84.5% (95% CI 77.8% – 89.8%),

**Negative predictive value **= 9108/9189 = 99.1% (95% CI 98.9% – 99.3%)

**LR (positive smear microscopy) **= 235.1 (95% CI 155.8 – 354.3)

**LR (negative smear microscopy) **= 0.38 (95% CI 0.32 – 0.45)

**Table 2:** Relation of colony forming units (CFUs) on culture to sensitivity of smear microscopy in 212 sputum samples

	**Quantitative sputum smear results**						
**Quantitative sputum culture results**	**< 10 afb/100 field**	**1+ (10–99afb/100 field)**	**2+ (1–10 afb/field)**	**3+ (> 10 afb/field)**	**Neg**	**Total**	**SM pos %**

**< 20 CFU**	4	6	1	1	41	53	**22.6**
**1+ (20–100 CFU)**	8	15	4	4	31	62	**50**
**2+ (> 100 CFU with discrete innumerable colonies)**	5	11	4	3	5	28	**82.1**
**3+ (> 100 CFU with confluent innumerable colonies)**	7	17	15	26	4	69	**94.2**

**Total**	**24**	**49**	**24**	**34**	**81**	**212**	**61.8**

A total of 103 (4.6%) subjects were sputum culture positive for MTB, 27 (1.2%) were sputum culture positive for NTM and 2 (0.1%) sputum culture positive for both MTB and NTM. Of the 103 subjects sputum culture positive for MTB, 40 (70%) had one positive sputum culture, 30 (29%) had two positive sputum cultures, and 33 (32%) had three positive sputum cultures. The incremental diagnostic yield was 74.8%, 9.7% and 15.5% for the first, second and third sputum culture respectively.

Sputum microscopy was positive for 57 (55%) of the 103 sputum culture positive subjects. Of the 57 smear positive patients, 12 (21%) had one positive smear, 14 (25%) had two positive smears and 31 (54%) had three positive smears. Forty seven patients has culture positive and smear negative results, of whom 31 (~66.0%) had one positive culture, 12 (25.5%) had two positive cultures and 4 (8.5%) had three positive cultures (Table 3). Most of the patients (22/31) who had one positive culture had growth of < 20 colonies, while most of those who had growth on all the three slants had a growth of 1+. Among seven sputum microscopy positive/sputum culture negative subjects, five were on TB treatment at the time sputum samples were submitted (Table 4). The incremental diagnostic value was 92.1%, 1.8% and 7.1% for the first, second and third smears, respectively.

**Table 3:** Sputum culture results in patients with negative sputum smears

No. pos sputum cultures	Quantitative sputum culture result			
	< 20 CFU	20–100 CFU	> 100 CFU	Total

1	22	6	2	30*
2	6	3	3	12
3	1	3	0	4

Total number	29	12	6	46

* 31 patients had one culture positive slant; however growth on one slant could not be estimated

**Table 4:** Clinical, radiological and microbiological findings of 7 patients who had smear positive, culture negative results

**Case**	**Smear 1**	**Smear 2**	**Smear 3**	**Clinical features**	**Conclusion**
1	**1+ **(10 – 99 AFB/100 oil fields)	**1+ **(10 – 99 AFB/100 oil fields)	3 AFBs	Cavity on CXR; patient treated for TB and responded	TB
2	**1+ **(10 – 99 AFB/100 oil fields)	Negative	Negative	Patient clinically well. Produced a total of 6 sputum specimens with only one positive smear	Not TB
3	**1+ **(10 – 99 AFB/100 oil fields)	**1+ **(10 – 99 AFB/100 oil fields)	Negative	Abdominal TB samples taken after TB Rx for 2 months.	TB
4	3AFBs/100 oil fields	Negative	Negative	Patient clinically well.	Not TB
5	6 AFBs/100 oil fileds	2 AFBs/100 oil fields	Negative	Fever and peritonitis with ascites by ultrasound, CXR normal, samples taken after 2 months of treatment.	TB
6	8 AFBs/100 oil fields	Negative	Negative	Prolonged cough and fever, CXR consistent with TB. Treated for TB and improved remarkably.	TB
7	(> 10 AFB/oil field)	Negative	Negative	Culture positive pulmonary TB, repeat samples taken after 2 months of treatment	TB

## Discussion

The data presented here on active TB screening of HIV-infected subjects with CD4 counts > 200 and suspect TB confirm that sputum microscopy is an insensitive tool for TB diagnosis in this setting. In addition the data show that the sensitivity of sputum microscopy is related to the bacterial burden in the positive sputum culture. The high levels of specificity, and positive predictive value of sputum microscopy in this study indicates that a positive test is useful in predicting the presence of pulmonary tuberculosis in HIV-infected individuals. The positive likelihood ratio of the sputum microscopy (235) suggests that a positive test almost rules in the diagnosis. However the negative LR is indicates that the probability of TB is only reduced by half in the face of a negative smear.

The level of sensitivity in the present study (61.8%) is higher than that found in Kenya (33%) [12], another HIV endemic area, which could be do to the fact that sputum samples in our study were concentrated before examination and possible differences in the degree of immuno-suppression between the studied populations. Eligibility for the study required a CD4 count > 200, a BCG scar, and no evidence of active TB. Thus the population tested may have had different characteristics to those of a general population of HIV positive individuals.

Despite the relatively higher sensitivity seen in our study, almost three quarters of patients with < 20 CFU would be missed by sputum microscopy. The consequences for this are several, including i) delayed or misdiagnosed, contributing to delayed treatment and increased morbidity and mortality rates [13-15]. For example, in the SIMHEALTH 611 study, 60% of the patients in whom the diagnosis of TB was missed, were being treated for non-specific pneumonia prior to death [16], ii) continued spread of TB to up to 20% of contacts [17], iii) underestimation of rates of disease and iv) inadequate exclusion of active TB in patients being considered for isoniazid preventive therapy [18]. These facts coupled with the reality that smear-negative TB cases have a poor prognosis [19] highlight the need for urgent measures to increase detection of smear negative TB cases and to raise the sensitivity of SM. Several laboratory methods have been tried such as sedimentation with either AFB phenol ammonium sulfate [20] or sodium hypochlorite [21], use of chitin in mucus digestion [22] and concentration by centrifugation [23],but most of these have limited testing under field conditions.

Most of the patients who had culture positive and smear negative result had discordant culture results. Majority of these patients had only one positive slant that yielded less than 20 colonies per slant, with only a few with three positive slants, which in many cases had a growth of 1+. However, there was no clear pattern of growth for those that yielded two positive slants.

We observed seven cases that had smear positive and culture negative results. Five of these cases were on anti-TB therapy, and it is likely that bacilli seen on the smears were not viable. The other two patients had each only one smear positive result, one had 3 AFB and the other had 1+, and is likely to be due to laboratory contamination. This is due to the fact that they had no signs or symptoms of tuberculosis and subsequently had good clinical outcome without TB therapy. Although the exact causes of these contamination in our laboratory was not identified, it may have resulted from either specimen mix-up, contamination of specimens or reagents with environmental mycobacteria in water, use of single-reagent delivery systems for multiple specimens or contamination in the cabinet area arising from sharing of the facility with several other laboratory workers of different projects. The significant diagnostic yield of the third smear found in this study differs from most other reports and may be a reflection of the particular method of sputum collection used in our protocol. The relatively high diagnostic value of the third smear is surprising because a recent systematic review of 37 studies show the third specimen lead to increase in sensitivity by 3.1% (95% CI, 2.1 to 4.2%) [24]. The second specimen gave surprisingly low incremental yield for both smears and cultures. This scenario may reflect poor instructions for specimens collected at home or very good instructions for the spot specimens and might also be due to mislabeling of the sequence by patients or by improved cough technique by the time of the third specimen. In our study, two samples for sputum microscopy and one for sputum culture would only detect TB in 76 (73.8%) of the 103 cases. However the problem of requesting three specimens would be that some patients may drop out of the diagnostic pathway between submitting specimens and being offered treatment [25]. This problem could be minimized by requesting two smears on the first visit [26] and by providing patient education coupled with collaboration between treatment supporters, health workers and community members in supervising TB patients [27].

## Conclusion

In summary we have shown that sputum microscopy will only detect slightly more than half of sputum culture positive cases of HIV-associated pulmonary tuberculosis in the setting of active case finding, that sensitivity is related to organism burden and that sputum culture are required for the optimal active case finding of HIV-associated TB.

## Competing interests

The author(s) declare that they have no competing interests.

## Authors' contributions

All authors conceived the study. MM, TL and WW were responsible for the laboratory part of the study. LM, RW, JL MB, KP and CFR were responsible for the clinical part of the study. All authors helped to conceptualize ideas and interpreted findings, and reviewed drafts of the manuscript.

## Pre-publication history

The pre-publication history for this paper can be accessed here:


